# In Vitro Reduction of Extractable Zearalenone and Screening of Tentative Transformation Products by *Metschnikowia pulcherrima* KKP 1368 Under Selected Buffered pH Conditions Relevant to the Porcine Gastrointestinal Tract

**DOI:** 10.3390/toxins18050214

**Published:** 2026-05-01

**Authors:** Krzysztof Waśkiewicz, Michał Dąbrowski, Michał Łuczyński, Marcin Wróbel, Łukasz Zielonka

**Affiliations:** 1 Department of Research and Development, Chemprof, Gutkowo 54B, 11-041 Olsztyn, Poland; krzysztof.waskiewicz@chemprof.pl (K.W.); michal.luczynski@chemprof.pl (M.Ł.); marcin.wrobel@chemprof.pl (M.W.); 2 Department of Veterinary Prevention and Feed Hygiene, Faculty of Veterinary Medicine, University of Warmia and Mazury in Olsztyn, Oczapowskiego 13, 10-718 Olsztyn, Poland; michal.dabrowski@uwm.edu.pl

**Keywords:** zearalenone (ZEN), yeast biomass, *Metschnikowia pulcherrima*, bioadsorption, biotransformation, LC-MS-QTOF, in vitro model

## Abstract

Zearalenone (ZEN) is an estrogenic mycotoxin produced by *Fusarium* spp. and commonly found in cereals and feed materials. This study evaluated the ability of *Metschnikowia pulcherrima* KKP 1368 biomass to reduce extractable ZEN under controlled buffered pH conditions (pH 3.50 and 7.00) selected as simplified conditions relevant to the porcine gastrointestinal environment. ZEN was quantified by LC-MS/MS, whereas LC-MS-QTOF was used as a qualitative/semi-quantitative screening approach for tentatively assigned transformation-related features. In the presence of yeast biomass, extractable ZEN was already lower than in the corresponding controls at the first sampling point, indicating a rapid biomass-associated effect. After 12 h, reductions relative to the corresponding controls reached 63.0% at pH 3.50 (*p* < 0.0001) and 51.6% at pH 7.00 (*p* = 0.0001). ZEN remained stable in control samples, and the strain remained viable under both pH conditions throughout incubation. LC-MS-QTOF detected several tentatively assigned features consistent with zearalanone, zearalenone-14-glucuronide, and zearalenol O-glucoside; these assignments require confirmation with authentic standards. Overall, *M. pulcherrima* KKP 1368 reduced extractable ZEN in a simplified buffered in vitro system, probably through rapid adsorption/reduced extractability and possible biotransformation. Further studies using biomass fractions or inactivated biomass, mass-balance experiments, authentic standards, and toxicological assays are needed to clarify the relative contribution of adsorption and transformation and to assess the practical relevance of this approach.

## 1. Introduction

Zearalenone (ZEN) is a resorcylic acid lactone mycotoxin produced mainly by *Fusarium* spp. and commonly reported in cereals and cereal-derived feedstuffs [1,2,3]. Because its molecular structure allows interaction with estrogen receptors, ZEN is considered an estrogenic endocrine-disrupting compound [3,4]. Pigs are particularly sensitive to ZEN; exposure has been associated with hyperestrogenic signs and reproductive disorders, including vulvar swelling, impaired fertility, pseudopregnancy, ovarian alterations, reduced litter size, and effects on male reproductive parameters [3,5].

ZEN is therefore relevant for feed safety and risk management in swine production. Recent industry monitoring data indicate that ZEN is detected in feed materials across several world regions; however, such annual survey data are best interpreted as evidence of broad occurrence rather than as proof of a long-term global increase [6]. In the European Union, Commission Regulation (EU) 2023/915 sets maximum levels for ZEN in selected food products, while Commission Recommendation 2006/576/EC provides guidance values for products intended for animal feeding, including complete and complementary feedstuffs for piglets, young gilts, sows, and finishing pigs [7,8].

From a practical feed perspective, any yeast-derived preparation intended for use as a feed additive in the European Union would require assessment and authorisation under the feed-additive regulatory framework [9]. Dietary strategies designed to lower mycotoxin exposure before intestinal absorption are relevant to intestinal health in monogastric animals; these include adsorbing agents as well as biotransforming agents [10]. For ZEN specifically, mitigation remains challenging because the compound is chemically stable and may resist conventional food and feed-processing methods [3,11,12]. Adsorbents are widely used, but ZEN is generally less efficiently adsorbed than some other mycotoxins, and non-specific mineral binders may interact with nutrients or micronutrients [13].

Biological approaches based on microorganisms, their cell walls, or cell-free enzymatic fractions have therefore received increasing attention. Bacteria, including lactic acid bacteria and species of *Lactobacillus* and *Bacillus*, have been reported to bind or transform ZEN under different in vitro conditions [14,15,16]. Yeast-derived materials are also attractive because cell-wall polysaccharides may contribute to rapid adsorption on the cell surface, whereas intracellular or extracellular enzymes may participate in toxin transformation [17]. Importantly, the practical relevance of such approaches does not necessarily depend on survival or proliferation of viable yeast cells in the gastrointestinal tract; inactivated biomass or cell-derived fractions may also contribute through sorption and potential residual or released enzymatic activities. Controlled in vitro studies are therefore useful as an initial step to separate pH-dependent biomass–toxin interactions from the complexity of the complete digestive environment.

Among non-conventional yeasts, *Metschnikowia pulcherrima* has attracted interest because of its ecological traits and biotechnological potential [18]. The species is commonly associated with fruits, flowers, grape must, and other plant-derived substrates, and it is known for producing pulcherriminic acid, which chelates ferric ions and may contribute to antagonistic activity against other microorganisms [18]. Although *Saccharomyces cerevisiae* and its cell-wall fractions have been studied more extensively for mycotoxin adsorption, less is known about non-conventional yeasts such as *M. pulcherrima* in the context of ZEN mitigation [17,18].

The present study evaluated the ability of *M. pulcherrima* KKP 1368 biomass to reduce extractable ZEN under selected buffered pH conditions (pH 3.50 and 7.00) chosen as simplified values relevant to the porcine gastrointestinal environment. ZEN concentrations were quantified by LC-MS/MS, whereas LC-MS-QTOF was used as a qualitative/semi-quantitative screening tool for tentatively assigned transformation-related features. The aim was to determine whether this strain can reduce extractable ZEN under controlled conditions and to provide a basis for future studies using inactivated biomass, biomass fractions, mass-balance experiments, and more complex feed or gastrointestinal models.

## 2. Results

### 2.1. LC-MS/MS Method Performance

The analytical method was developed using six-point extraction calibration curves covering the analyte concentration range of 30.625–980 ng/mL. The correlation coefficients were R = 0.9967 and R = 0.9952 for the six-point extraction calibration curves prepared at pH 3.50 and 7.00, respectively, confirming acceptable linearity across the tested range. Based on a signal-to-noise ratio (S/N) of 10:1, the LOQ was 1 ng/mL, while the LOD was 0.3 ng/mL. The relative standard deviation (RSD%) was estimated at three concentration levels selected from the calibration range (30.625, 245, and 980 ng/mL), and was 3.84%, 4.13%, and 3.82%, respectively.

### 2.2. Reduction of Extractable Zearalenone During Incubation

ZEN concentrations measured by targeted LC-MS/MS are presented in Table 1. In the controls, ZEN remained stable throughout the 12 h experiment at both pH values, with mean concentrations ranging from 536 to 558 ng/mL at pH 3.50 and from 541 to 553 ng/mL at pH 7.00. In contrast, in the presence of *M. pulcherrima* KKP 1368 biomass, extractable ZEN was already markedly lower than in the corresponding control at the first sampling point (0 h), reaching 262 ng/mL at pH 3.50 and 337 ng/mL at pH 7.00. This immediate difference indicates a rapid biomass-associated reduction in extractable ZEN rather than a gradual time-dependent process alone.

After this initial decrease, the concentration profile was not strictly linear. At pH 3.50, mean ZEN concentration in yeast-biomass samples increased from 262 ng/mL at 0 h to 345 ng/mL at 3 h and then decreased to 203 ng/mL at 12 h. At pH 7.00, a smaller transient increase was observed between 0 and 1 h, followed by a gradual decrease to 262 ng/mL at 12 h. Relative to the corresponding controls, the reduction after 12 h reached 63.0% at pH 3.50 and 51.6% at pH 7.00. Thus, the observed profile is consistent with rapid adsorption/reduced extractability at the beginning of contact, followed by further changes during incubation.

### 2.3. Yeast Viability After Incubation

**Figure 1:**
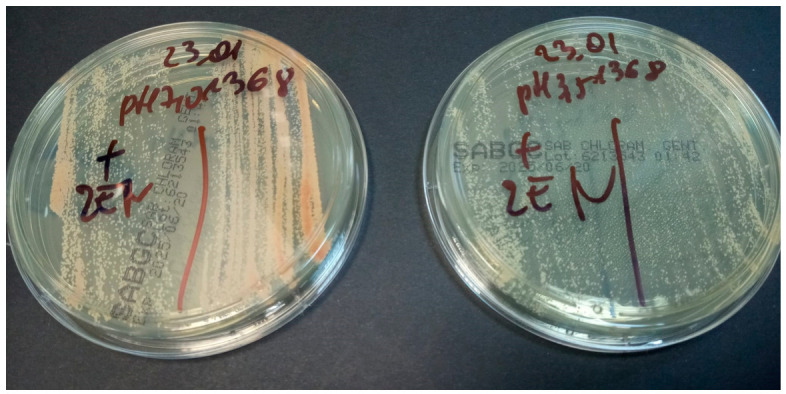
Representative Sabouraud plates taken after 12 h of incubation from ZEN-containing samples. Cultures incubated at pH 7.00 are shown on the (**left**), and those at pH 3.50 are shown on the (**right**).

After incubation, *M. pulcherrima* KKP 1368 remained culturable on Sabouraud agar under both pH conditions. Visible colony growth was observed from samples incubated with ZEN at pH 3.50 and pH 7.00, and no obvious inhibition of colony development by ZEN was observed under the tested conditions (Figure 1). This result indicates that the experimental conditions did not eliminate viable yeast cells during the 12 h contact period.

### 2.4. Statistical Evaluation of the Effects of pH, Sample Type, and Time

A three-way analysis of variance (pH × sample type × time) was applied to the LC-MS/MS dataset, with independent biological replicates at each time point. The full ANOVA summary is provided in Table 2. The analysis showed significant effects of pH, sample type, and time, as well as significant pH × sample type and sample type × time interactions, whereas the three-way interaction was not significant. These findings indicate that the reduction of extractable ZEN depended on the presence of yeast biomass and incubation conditions rather than on spontaneous instability of ZEN in the corresponding buffered controls. The assumptions of ANOVA were satisfied: in each cell of the arrangement, the Shapiro–Wilk test showed no deviations from normality, and the Brown–Forsythe version of Levene’s test confirmed homogeneity of variances.

### 2.5. LC-MS-QTOF Screening in Positive Ionisation Mode

**Figure 2:**
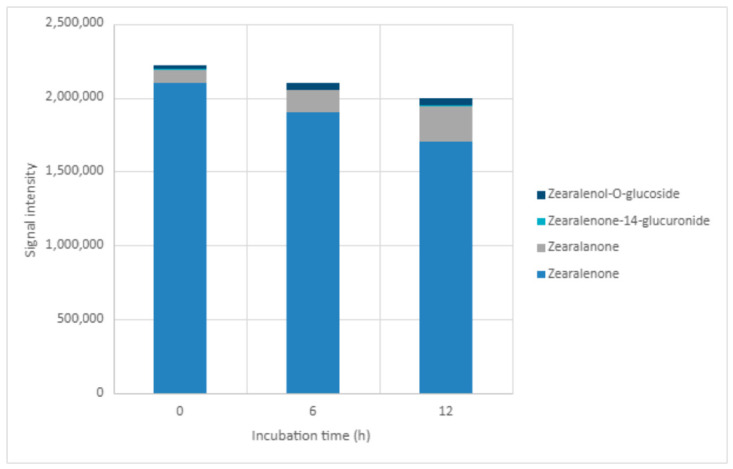
Distribution of peak areas for ZEN and tentatively assigned transformation-related features during incubation at pH 3.50 in positive ionisation mode.

**Figure 3:**
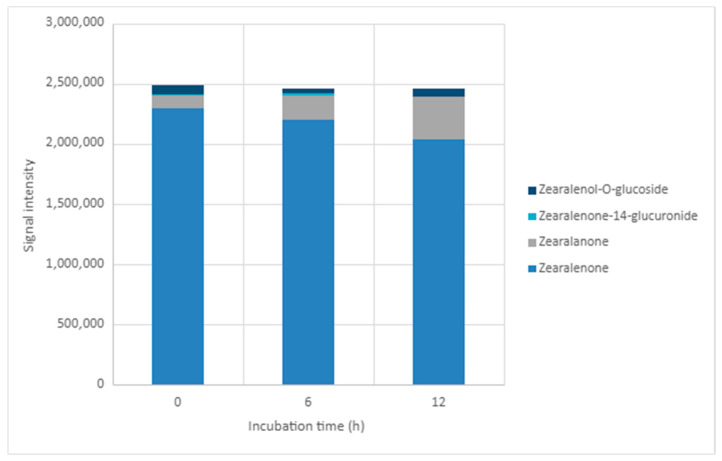
Distribution of peak areas for ZEN and tentatively assigned transformation-related features during incubation at pH 7.00 in positive ionisation mode.

LC-MS-QTOF was used as a qualitative/semi-quantitative screening approach to monitor changes in peak areas for ZEN and tentatively assign transformation-related features. Because authentic standards and compound-specific calibration curves were not available for all proposed products, peak areas were not converted into concentrations and should not be directly compared with LC-MS/MS-derived reduction percentages. In positive ionisation mode, signals consistent with ZEN, zearalanone, zearalenone-14-glucuronide, and zearalenol O-glucoside were monitored at 0, 6, and 12 h under acidic (pH 3.50) and neutral (pH 7.00) conditions (Table 3; Figure 2 and Figure 3; Supplementary Figures S1 and S2). The corresponding annotation parameters, including formula, retention time, adduct, observed and theoretical *m*/*z* values, mass error, basis for annotation, and annotation status, are summarised in Supplementary Table S1.

At pH 3.50, the ZEN peak area decreased from 2.10 × 10^6^ at 0 h to 1.70 × 10^6^ at 12 h, while the zearalanone-related signal increased from 9.03 × 10^4^ to 2.45 × 10^5^. At pH 7.00, the ZEN signal decreased from 2.29 × 10^6^ to 2.03 × 10^6^, and the zearalanone-related signal increased from 1.06 × 10^5^ to 3.55 × 10^5^. The feature tentatively assigned to ZEN-14-glucuronide was weak and sporadic at pH 3.50 and remained low at pH 7.00. The feature tentatively assigned to zearalenol O-glucoside was detected at all sampling points in both pH conditions, with peak areas ranging from 2.00 × 10^4^ to 4.94 × 10^4^ at pH 3.50 and from 4.72 × 10^4^ to 7.71 × 10^4^ at pH 7.00. Overall, the QTOF profile supports the presence of transformation-related signals, especially an increasing zearalanone-related feature, but these assignments should be considered tentative until confirmed with authentic standards.

### 2.6. LC-MS-QTOF Screening in Negative Ionisation Mode

**Figure 4:**
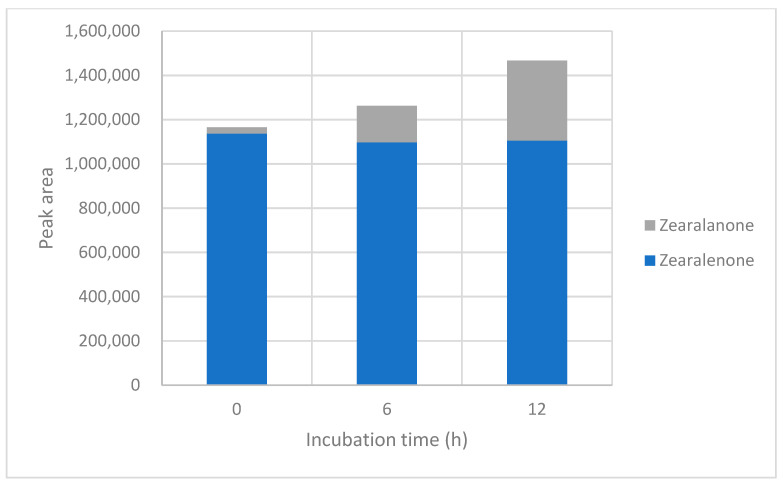
Distribution of peak areas for ZEN and the tentatively assigned zearalanone-related feature during incubation at pH 3.50 in negative ionisation mode.

**Figure 5:**
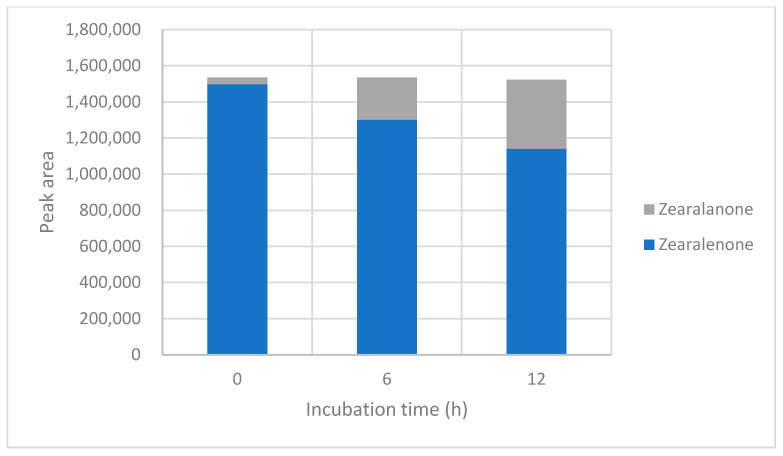
Distribution of peak areas for ZEN and the tentatively assigned zearalanone-related feature during incubation at pH 7.00 in negative ionisation mode.

In negative ionisation mode, ZEN and a feature tentatively assigned to zearalanone were detected (Table 4; Figure 4 and Figure 5; Supplementary Figures S3 and S4). The absence of conjugated features in negative ionisation should be interpreted with caution, as detectability in LC-MS-QTOF screening depends on ionisation efficiency, adduct formation, abundance, and matrix-dependent response. At pH 3.50, the ZEN signal remained relatively stable across the three time points (1.14 × 10^6^ to 1.10 × 10^6^), whereas the zearalanone-related signal increased from 2.86 × 10^4^ to 3.63 × 10^5^. At pH 7.00, the ZEN signal decreased from 1.50 × 10^6^ to 1.14 × 10^6^, and the zearalanone-related signal increased from 3.76 × 10^4^ to 3.83 × 10^5^. These semi-quantitative data are consistent with the positive-ionisation results in indicating a time-dependent increase in the zearalanone-related feature, but they do not provide an independent quantitative mass balance for ZEN transformation.

## 3. Discussion

The present study was designed as a controlled screening of ZEN interactions with *M. pulcherrima* KKP 1368 biomass under selected buffered pH conditions. The results show that the presence of yeast biomass markedly reduced extractable ZEN, whereas ZEN remained stable in the corresponding controls. This confirms that the changes observed in yeast-biomass samples were not caused by spontaneous degradation of ZEN in the buffer, but by interactions between the toxin and the yeast-derived material.

The concentration profile indicates that the process should not be interpreted as a simple linear biodegradation curve. Extractable ZEN was already lower in yeast-biomass samples at the first sampling point, which is consistent with a rapid biomass-associated effect. This initial decrease was followed by a partial increase during the first hours of incubation and then by a further decrease up to 12 h. Such a pattern is compatible with rapid adsorption to cell-wall components, reduced extractability of cell-bound toxin, and subsequent redistribution between free and biomass-associated fractions. Therefore, the term ‘reduction of extractable ZEN’ is more appropriate than direct attribution of the whole effect to biodegradation.

Rapid binding of ZEN by yeast biomass has been described mainly for *Saccharomyces*-based materials and isolated yeast cell-wall fractions. Cell-wall β-glucans and related polysaccharide structures are considered important binding sites, and the equilibrium between free and bound toxin may depend on pH, toxin concentration, cell-wall structure, and the physicochemical state of the biomass [19,20,21,22,23]; for a broader review of source-dependent β-glucan structure and function, see Choi et al. [24]. The immediate reduction observed in the present experiment is in line with these reports. However, because the study did not include a pellet/supernatant mass balance or a dedicated recovery experiment for cell-bound ZEN, the relative contribution of adsorption and reduced analytical recovery cannot be quantified from the current data.

Compared with reports on *Saccharomyces cerevisiae* biomass, isolated yeast cell-wall fractions, and other yeast-derived materials, the reductions observed here fall within the range described for yeast-based ZEN-mitigation systems [20,21,22,23,24,25]. At the same time, direct comparison between studies remains only approximate because published experiments differ in toxin concentration, biomass dose, incubation time, pH, sample matrix, and analytical workflow. Therefore, the present findings should not be interpreted as evidence that *M. pulcherrima* KKP 1368 is inherently superior to *S. cerevisiae* or other yeasts, but rather as evidence that this non-conventional yeast is a promising source of biomass for further controlled ZEN-reduction studies.

Although direct comparisons across yeast studies remain difficult because strain identity, biomass form, toxin concentration, incubation conditions, and analytical endpoints vary substantially, the 12 h reductions observed here can be set within the broader context of yeast-based ZEN mitigation. In an in vitro study, Chlebicz and Śliżewska reported that Saccharomyces cerevisiae strains reduced ZEN concentration by an average of 34.45% after 6 h, with an additional 11.59% over the next 6 h, corresponding to approximately 46% after 12 h and 52% after 24 h [14]. More broadly, previous studies on S. cerevisiae biomass, yeast cell-wall preparations, and other microbial systems indicate that both adsorption and biotransformation may contribute to the apparent reduction in extractable ZEN [20,21,22,23]. By contrast, Zhang et al. reported complete degradation of ZEN by S. cerevisiae only after 48 h, illustrating how strongly apparent efficiency depends on the experimental design and the endpoint selected [25]. Against this background, the 12 h reductions observed here for M. pulcherrima KKP 1368 (63.0% at pH 3.50 and 51.6% at pH 7.00) fall within the range reported for yeast-based systems, but they do not justify claims of superiority over S. cerevisiae or other yeasts in the absence of a direct head-to-head comparison [14,20,21,22,23,25].

In addition to the decrease in extractable ZEN, LC-MS-QTOF screening revealed several features tentatively assigned to ZEN-related transformation products. These observations suggest that biotransformation may also contribute to the overall profile. Nevertheless, the QTOF data should be interpreted as qualitative/semi-quantitative screening results. The peak areas were not converted to concentrations because authentic standards and compound-specific calibration curves were unavailable for all tentatively assigned products. Consequently, LC-MS-QTOF peak-area trends should not be directly compared with the quantitative LC-MS/MS percentages of ZEN reduction. Differences between both datasets are expected because signal intensity in QTOF analysis is influenced by ionisation efficiency, adduct formation, matrix effects, and compound-specific response factors.

Several microbial and enzymatic mechanisms of ZEN transformation have been reported previously, including lactone-ring hydrolysis, cleavage, reduction, and conjugation [12,25,26,27,28,29,30,31,32]. In the present study, a zearalanone-related signal was among the most relevant tentatively assigned features. Zearalanone can be regarded as a reduced ZEN-related compound, but its toxicological interpretation requires caution because estrogenic potency varies substantially among ZEN derivatives and is strongly structure-dependent [3,4]. Therefore, the occurrence of a zearalanone-related signal should not be considered sufficient evidence of detoxification without confirmation using authentic standards and biological activity assays.

The tentatively assigned conjugated features also require careful interpretation. Glucuronide and glucoside derivatives of ZEN or zearalenols may show lower apparent estrogenic activity in some in vitro systems, but masked or conjugated mycotoxins can be hydrolysed under gastrointestinal or microbial conditions and may therefore contribute to internal exposure [33,34,35,36,37,38]. The low and inconsistent signal assigned to ZEN-14-glucuronide in the present dataset does not allow conclusions about the glucuronidation capacity of *M. pulcherrima* KKP 1368. Similarly, the zearalenol O-glucoside-related signal should be treated as a tentative annotation until confirmed by authentic standards and/or targeted MS/MS fragmentation in future work.

The lack of ZEN-14-glucuronide- and zearalenol O-glucoside-related signals in the negative-ionisation dataset should not be interpreted as proof of their absence. In the present LC-MS-QTOF workflow, detectability depended on compound-specific ionisation efficiency, adduct formation, signal abundance, and matrix-dependent response. Accordingly, features tentatively detectable in the positive mode may remain below the practical detection threshold in the negative mode, particularly when present at low relative abundance. This limitation is consistent with the qualitative/semi-quantitative character of the screening approach used here and supports cautious interpretation of these annotations.

The buffered model used in this study was intentionally simplified. It was not designed to reproduce the full biochemical complexity of the porcine gastrointestinal tract, where digestive enzymes, bile salts, feed matrix components, microbiota, transit time, and regional pH gradients may all affect both ZEN fate and yeast-derived biomass. Instead, the aim was to isolate the effect of yeast biomass under two controlled pH conditions relevant to selected gastrointestinal environments. From a practical feed perspective, further development of a yeast-derived product would also need to comply with European Union authorisation procedures for feed additives [9]. Such a product would not necessarily rely on survival and proliferation of viable *M. pulcherrima* cells in vivo, but could involve inactivated biomass or cell-derived preparations acting through sorption and, depending on processing, residual or released enzymatic activities.

Taken together, the results support further evaluation of *M. pulcherrima* KKP 1368 as a source of yeast-derived material for ZEN mitigation, but they do not yet establish complete biodegradation or detoxification. Future studies should include inactivated biomass, cell-free extracts, separated cell-wall fractions, pellet/supernatant mass-balance experiments, matrix-matched recovery controls, confirmation of transformation products with authentic standards, and toxicological assays to determine whether the observed reduction in extractable ZEN translates into a real decrease in estrogenic activity.

## 4. Conclusions

Under selected buffered pH conditions, *M. pulcherrima* KKP 1368 biomass reduced extractable ZEN, with 12 h reductions of 63.0% at pH 3.50 and 51.6% at pH 7.00 relative to the corresponding controls. Because extractable ZEN was already lower in yeast-biomass samples at the first sampling point, the observed effect should be interpreted primarily as a rapid biomass-associated reduction, probably involving adsorption and/or reduced extractability of cell-bound ZEN, with a possible contribution of biotransformation during incubation. The strain remained culturable under both tested pH conditions.

LC-MS-QTOF screening revealed tentatively assigned ZEN-related features, including zearalanone-, ZEN-14-glucuronide-, and zearalenol O-glucoside-related signals. These qualitative/semi-quantitative data support the hypothesis that transformation-related processes may occur, but they do not by themselves demonstrate detoxification. Further studies using inactivated biomass, cell-free fractions, pellet/supernatant mass balance, matrix-matched recovery controls, authentic standards, and toxicological assays are required before practical application as a yeast-derived ZEN-mitigation strategy can be considered.

## 5. Materials and Methods

### 5.1. Chemicals, Buffers, and Reagents

Zearalenone (ZEN) was purchased from Cayman Chemical (Ann Arbor, MI, USA), and a working solution was prepared in ethanol at approximately 100 μg/mL. A 0.1 M phosphate buffer was prepared from KH_2_PO_4_ in ultrapure water and adjusted to pH 3.50 or 7.00 using H_3_PO_4_ and KOH (Chempur, Piekary Śląskie, Poland). The two pH values were selected to provide controlled buffered conditions relevant to acidic and near-neutral gastrointestinal environments, rather than to reproduce the full biochemical complexity of the porcine gastrointestinal tract.

### 5.2. Yeast Strain, Culture Conditions, and Inoculum Preparation

The yeast strain *Metschnikowia pulcherrima* KKP 1368 was obtained from the Institute of Biotechnology of the Agricultural and Food Industry in Warsaw, Poland. To propagate the strain, it was streaked onto Sabouraud agar supplemented with chloramphenicol (Oxoid Ltd., Basingstoke, Hampshire, UK). The plates were incubated at 28–30 °C for 7 days. After incubation, a single colony was picked with a sterile inoculating loop and transferred into a flask containing Sabouraud broth (Oxoid Ltd., Basingstoke, Hampshire, UK). The flask was placed in a shaking incubator (New Brunswick Scientific, Edison, NJ, USA) at 150–200 rpm and 28–30 °C and incubated for 18–24 h. After this period, the optical density of the suspension was measured at 600 nm (OD_600_) using a Sunrise absorbance reader (Tecan, Grödig, Austria). Based on the obtained values, the culture was diluted in sterile saline (0.9% NaCl; POLPHARMA S.A., Starogard Gdański, Poland) to obtain a final suspension concentration of 10^7^ colony-forming units per millilitre (CFU/mL).

### 5.3. Incubation Design, Controls, and Sample Extraction

The incubation experiment was designed as a controlled contact assay between ZEN and yeast biomass under selected buffered pH conditions. A 1 mL aliquot of the prepared yeast suspension was added to a tube containing 8.9 mL of buffer and 0.1 mL of ZEN working solution at 98 μg/mL, resulting in a nominal initial ZEN concentration of 980 ng/mL before extraction. The mixtures were incubated at 37 °C and sampled immediately after mixing (0 h) and after 1, 3, 6, and 12 h. The 0 h samples were collected immediately after combining the reaction components and therefore represent the initial extractable ZEN level after contact with yeast biomass and acetonitrile extraction. The 12 h endpoint was selected as a short-term screening period to follow early interactions between ZEN and yeast biomass under controlled pH conditions. Collected samples were extracted with acetonitrile (Merck KGaA, Darmstadt, Germany) at a 1:1 sample-to-solvent volume ratio to stop the incubation and recover extractable ZEN, including ZEN potentially associated with yeast biomass. After extraction, samples were filtered through 0.22 μm syringe filters into chromatographic vials and analysed. Each time point was prepared as three independent biological replicates, and the experiment was conducted separately in buffers at pH 3.50 and 7.00. Control samples were prepared in the same way, except that 1 mL of buffer was added instead of the yeast suspension. Because no separate pellet/supernatant mass balance was performed, LC-MS/MS results are reported as extractable ZEN under the applied extraction conditions.

### 5.4. Yeast Viability Assessment

Samples for viability assessment were prepared in parallel using the same incubation conditions, except that the final acetonitrile extraction step was omitted. Additional samples without ZEN were included to assess whether the mycotoxin affected strain culturability under the tested conditions. After incubation, samples were plated onto Sabouraud agar supplemented with chloramphenicol (Oxoid, UK), and colony growth was evaluated visually as a qualitative confirmation of yeast survival/culturability.

### 5.5. Calibration Solutions and Six-Point Extraction Calibration Curves

The stock solution for calibration was prepared by mixing 0.2 mL of the initial ZEN working solution with 9.8 mL of buffer and 10 mL of acetonitrile. Serial dilutions of this mixture were then prepared in buffer/acetonitrile (1:1, *v*/*v*) to obtain six calibration levels: 30.625, 61.25, 122.5, 245, 490, and 980 ng/mL. Two six-point extraction calibration curves were prepared, one for each buffer pH. These calibration solutions represented the buffer/acetonitrile extraction matrix and did not contain yeast biomass; therefore, the quantified values should be interpreted as extractable ZEN recovered under the applied extraction procedure. The preparation scheme for the reaction mixtures and the highest calibration solution is provided in Table 5.

### 5.6. LC-MS/MS Quantification of Zearalenone

Targeted quantification of extractable ZEN was performed using an Agilent chromatographic system (Agilent Technologies, Inc., Santa Clara, CA, USA) comprising a G7167A multisampler, a G7104C quaternary gradient pump, a G7116A column oven, and a 6470 LC/TQ triple quadrupole mass spectrometer. Chromatographic separation was performed using a ZORBAX Eclipse Plus C18 column (2.1 × 50 mm, 1.8 μm; Agilent Technologies, Inc., Santa Clara, CA, USA) with gradient elution using 0.1% formic acid + 0.5 mM ammonium fluoride in water (Merck KGaA, Darmstadt, Germany) as mobile phase A and 0.1% formic acid + 0.5 mM ammonium fluoride in acetonitrile (Merck KGaA, Darmstadt, Germany) as mobile phase B. The flow rate was 0.250 mL/min, the injection volume was 5 μL, and the column temperature was kept at 40 °C. The mass spectrometer, equipped with an Agilent Jet Stream ESI source, was operated in positive ionisation mode. The ion source parameters included a nebulising gas temperature of 250 °C with a flow rate of 8 L/min, a nebuliser pressure of 30 psi, a sheath gas temperature of 350 °C with a flow rate of 12 L/min, and a capillary voltage of 3300 V. The gradient programme is detailed in Table 6, and the MRM scan parameters are listed in Table 7.

### 5.7. Validation of the LC-MS/MS Method

The LC-MS/MS method was validated for targeted quantification of ZEN under the conditions described above. Validation was performed using two separate six-point extraction calibration curves prepared in buffers at pH 3.50 and 7.00, covering six concentration levels: 30.625, 61.25, 122.5, 245, 490, and 980 ng/mL. Linearity of the response, method sensitivity based on empirically determined LOD and LOQ values after serial standard dilutions, and precision expressed as relative standard deviation (RSD) at three concentration levels (30.625, 245, and 980 ng/mL) were assessed.

### 5.8. LC-MS-QTOF Screening of Tentative Transformation-Related Features

Samples collected after 0, 6, and 12 h of incubation at pH 3.50 and 7.00 were analysed by LC-MS-QTOF as a qualitative/semi-quantitative screening approach for ZEN and tentatively assigned ZEN-related transformation features. After incubation, samples were extracted with acetonitrile (1:1, *v*/*v*) and filtered through 0.22 μm syringe filters into chromatographic vials, as described above. Chromatographic separation was performed on an Agilent 1260 Infinity III HPLC system (Agilent Technologies, Santa Clara, CA, USA) equipped with a 600 bar binary pump, a thermostatted autosampler, and a column oven. Separation was carried out on an Agilent InfinityLab Zorbax Eclipse Plus C18 Rapid Resolution HD column (2.1 × 50 mm, 1.8 μm) maintained at 40 °C. The flow rate was set at 0.4 mL/min, with an injection volume of 1 μL. Mobile phase A consisted of water with 0.1% formic acid and 5 mM ammonium formate, whereas mobile phase B consisted of methanol with 0.1% formic acid and 5 mM ammonium formate. The gradient elution programme is detailed in Table 8, with a total run time of 27 min.

Mass spectrometric detection was performed using an Agilent Revident Q-TOF mass spectrometer (Agilent Technologies, Santa Clara, CA, USA) equipped with a Dual ESI JetStream ion source. Data were collected in centroid mode using the All Ions acquisition method over an *m*/*z* range of 50–1000 in both positive and negative ionisation modes at a rate of 6 spectra/s. Collision energies of 0, 15, and 30 V were applied to obtain fragmentation information. Detailed source and acquisition parameters are listed in Table 9. Data processing was performed using MassHunter Qualitative Analysis (version 10, Agilent Technologies) with suspect-screening and untargeted workflows. Suspect screening used the Find-by-Formula algorithm with the Mycotoxins PCDL B.07.00 library, while untargeted feature detection used the Molecular Feature Extraction algorithm. Feature annotation was further supported by comparison with the PCDL Metlin_Metabolites_AM_PCDL database and by evaluation of accurate mass, isotope pattern, adduct formation, retention behaviour, and database/literature consistency. Only adducts with an absolute mass error not exceeding 5 ppm were retained in Supplementary Table S1. Although All Ions acquisition was performed, feature-specific product-ion assignments were not available for the tentative products; therefore, all ZEN-related products other than ZEN itself were reported as tentative transformation-related features. Because authentic standards and compound-specific calibration curves were not available for all tentative products, LC-MS-QTOF peak areas were used only for relative comparison of the same feature within the same ionisation mode and were not converted into concentrations. The LC-MS-QTOF annotation parameters for the monitored and tentatively assigned features are provided in Supplementary Table S1.

### 5.9. Statistical Analysis

Statistical analysis was conducted using STATISTICA 13.3 software (TIBCO Software Inc., Palo Alto, CA, USA). The LC-MS/MS dataset was analysed using a 2 × 2 × 5 factorial treatment arrangement: pH condition (pH 3.50 and pH 7.00) × sample type (control and yeast-biomass sample) × time (0, 1, 3, 6, and 12 h), with three independent biological replicates per cell (60 observations in total). The experimental unit was a single, independently prepared incubation tube. Because each sampling time was represented by a separate tube, the observations were treated as independent rather than repeated measurements. Normality was evaluated using the Shapiro–Wilk test, and homogeneity of variances was examined using Levene’s test in the median version (Brown–Forsythe modification). A three-way ANOVA was used as the primary analysis to assess the effects of pH condition, sample type, time, and their interactions. Means, standard deviations, test statistics, degrees of freedom, and *p*-values were reported where appropriate. The significance level was set at α = 0.05 (two-tailed).

## 6. Patents

The findings presented in this manuscript are related to Polish patent application P.455139 [WIPO ST 10/C PL455139], which covers aspects of the approach described herein. The application is currently under formal examination.

## Figures and Tables

**Table 1:** Mean ± SD ZEN concentrations in control and yeast-biomass samples measured by LC-MS/MS (*n* = 3).

ZEN Concentration (ng/mL; Mean ± SD, n = 3)				
	Control		Yeast-Biomass Sample	
Time (h)	pH = 3.50	pH = 7.00	pH = 3.50	pH = 7.00
0	536 ± 28	551 ± 5	262 ± 75	337 ± 42
1	547 ± 27	545 ± 11	319 ± 39	367 ± 22
3	558 ± 4	552 ± 4	345 ± 20	328 ± 23
6	553 ± 17	553 ± 9	248 ± 50	301 ± 43
12	548 ± 19	541 ± 3	203 ± 26	262 ± 34

**Table 2:** Three-way ANOVA results for the effects of pH, sample type, and time on LC-MS/MS ZEN concentrations.

Effect	df	F	*p*
**pH**	1, 40	7.38	0.010
**Sample type**	1, 40	997.44	<0.001
**Time**	4, 40	7.11	<0.001
**pH × sample type**	1, 40	7.46	0.009
**pH × time**	4, 40	1.35	0.267
**Sample type × time**	4, 40	6.20	<0.001
**pH × sample type × time**	4, 40	0.79	0.542

**Table 3:** Positive-ionisation LC-MS-QTOF screening results. Signals of ZEN and tentatively assigned transformation-related features are expressed as peak areas.

pH	Time (h)	ZEN	Zearalanone	ZEN-14-Glucuronide	Zearalenol O-Glucoside	Sum of Peak Areas
**3.50**	0	2,096,386	90,323	9212	20,048	2,215,970
**3.50**	6	1,901,120	149,949	0	45,931	2,097,001
**3.50**	12	1,698,701	244,524	4639	49,410	1,997,274
**7.00**	0	2,294,231	106,127	7797	77,066	2,485,221
**7.00**	6	2,197,118	205,161	14,627	47,248	2,464,154
**7.00**	12	2,032,705	355,136	7936	67,828	2,463,605

**Table 4:** Negative-ionisation LC-MS-QTOF screening results. Signals of ZEN and the tentatively assigned zearalanone-related feature are expressed as peak areas.

pH	Time (h)	ZEN	Zearalanone	Sum of Peak Areas
**3.50**	0	1,136,822	28,568	1,165,390
**3.50**	6	1,097,007	165,185	1,262,192
**3.50**	12	1,104,945	362,526	1,467,471
**7.00**	0	1,497,527	37,625	1,535,152
**7.00**	6	1,301,221	234,619	1,535,840
**7.00**	12	1,140,005	382,997	1,523,001

**Table 5:** Scheme of the preparation of reaction mixtures and the highest calibration solution before serial dilution, including nominal ZEN concentrations before and after acetonitrile extraction.

Sample Type	Buffer (mL) (pH 3.50/7.00)	ZEN Working Solution (mL)	Yeast Suspension (mL)	Nominal ZEN Concentration Before Extraction (ng/mL)	Acetonitrile (mL)	Nominal ZEN Concentration After Extraction (ng/mL)
**Control**	9.9	0.1	-	980	10	490
**Yeast-biomass sample**	8.9	0.1	1	980	10	490
Highest calibration level	9.8	0.2	-	1960	10	980

**Table 6:** Gradient programme of the mobile phases used for LC-MS/MS quantification. Phase A: water with 0.1% formic acid and 0.5 mM ammonium fluoride; Phase B: acetonitrile with 0.1% formic acid and 0.5 mM ammonium fluoride.

Time (min)	Phase A (%)	Phase B (%)
**0.00**	**80.0**	**20.0**
**1.00**	80.0	20.0
**4.00**	0.0	100.0
**7.00**	0.0	100.0
**7.01**	80.0	20.0
**15.00**	80.0	20.0

**Table 7:** Optimized MRM parameters for ZEN quantification.

Compound	Precursor Ion (*m*/*z*)	Product Ion (*m*/*z*)	Fragmentor (V)	Collision Energy (V)	Retention Time (min)	Ionisation Mode
**Zearalenone**	319.2	301.1	70	10	5.78	Positive
**Zearalenone**	319.2	283.1	70	10	5.78	Positive

**Table 8:** Gradient programme used for chromatographic separation in LC-MS-QTOF screening. Phase A: water with 0.1% formic acid and 5 mM ammonium formate; Phase B: methanol with 0.1% formic acid and 5 mM ammonium formate.

Time (min)	Phase A (%)	Phase B (%)
**0.00**	95.0	5.0
**1.00**	95.0	5.0
**15.00**	5.0	95.0
**21.00**	5.0	95.0
**21.01**	95.0	5.0
**27.00**	95.0	5.0

**Table 9:** Mass spectrometry source and acquisition parameters used for LC-MS-QTOF screening.

Parameter Group	Setting
**All Ions acquisition parameters**	*m*/*z* range: 50–1000
	Acquisition rate: 6.0 spectra/s
	Time per spectrum: 166.7 ms
**Ion source parameters**	Gas temperature: 250 °C
	Drying gas flow: 8 L/min
	Nebulizer pressure: 40 psi
	Sheath gas temperature: 350 °C
	Sheath gas flow: 12 L/min
	Capillary voltage: 4000 V
	Nozzle voltage: 0 V
	Fragmentor: 120 V
**Additional parameters**	Skimmer: 65 V
	Oct 1 RF Vpp: 400 V
	Quad Amu: 148

## Data Availability

The original contributions presented in this study are included in the article/Supplementary Material. Further inquiries can be directed to the corresponding author.

## Supplementary Materials

The following supporting information can be downloaded at: https://www.mdpi.com/article/10.3390/toxins18050214/s1, Table S1: LC-MS-QTOF annotation parameters for ZEN and tentatively assigned transformation-related features; Figure S1: heatmap of LC-MS-QTOF peak-area distribution in positive ionisation mode at pH 3.50; Figure S2: heatmap of LC-MS-QTOF peak-area distribution in positive ionisation mode at pH 7.00; Figure S3: heatmap of LC-MS-QTOF peak-area distribution in negative ionisation mode at pH 3.50; Figure S4: heatmap of LC-MS-QTOF peak-area distribution in negative ionisation mode at pH 7.00.

## Abbreviations

The following abbreviations are used in this manuscript:

ANOVA	analysis of variance
CFU	colony-forming units
ESI	electrospray ionisation
EU	European Union
HPLC	high-performance liquid chromatography
LC-MS/MS	liquid chromatography–tandem mass spectrometry
LC-MS-QTOF	liquid chromatography–quadrupole time-of-flight mass spectrometry
LOD	limit of detection
LOQ	limit of quantification
MRM	multiple reaction monitoring
OD600	optical density at 600 nm
PCDL	personal compound database and library
QTOF	quadrupole time-of-flight
RSD	relative standard deviation
SD	standard deviation
S/N	signal-to-noise ratio
ZEN	zearalenone
