# Supplementary material for: In Vitro Reduction of Extractable Zearalenone and Screening of Tentative Transformation Products by Metschnikowia pulcherrima KKP 1368 Under Selected Buffered pH Conditions Relevant to the Porcine Gastrointestinal Tract
Source: Toxins (Basel). 2026 May 1;18(5):214. doi: 10.3390/toxins18050214 (PMC13211369; doi:10.3390/toxins18050214)
Supplement: Supplementary file 1 [file toxins-18-00214-s001.zip › toxins-4242708-supplementary.pdf]

Table S1. LC-MS-QTOF annotation parameters for ZEN and tentatively assigned transformation-related features.

Theoretical m/z values were calculated for the specified adducts. Mass error was calculated as [(observed m/z − theoretical m/z) / theoretical m/z] × 10^6. Only adducts with an absolute mass error not exceeding 5 ppm were retained in the table. Feature-specific product-ion assignments were not available for the tentative transformation-related features; therefore, annotations other than ZEN should be regarded as tentative and were based on accurate mass, adduct formation, retention behaviour, and database/literature consistency.

Feature	Formula	Ion mode	RT (min)	Adduct	Obs. m/z	Theor. m/z	Error (ppm)	Annotation basis	Annotation status
ZEN	C18H22O5	ESI(+)	13.893	[M+H]+	319.1540	319.1540	0.00	Authentic standard; RT; exact mass/adduct	Confirmed by standard
ZEN	C18H22O5	ESI(+)	13.893	[M+NH4]+	336.1802	336.1805	-1.04	Authentic standard; RT; exact mass/adduct	Confirmed by standard
ZEN	C18H22O5	ESI(+)	13.893	[M+Na]+	341.1359	341.1359	-0.13	Authentic standard; RT; exact mass/adduct	Confirmed by standard
ZEN	C18H22O5	ESI(−)	13.857	[M−H]−	317.1402	317.1394	+2.37	Authentic standard; RT; exact mass/adduct	Confirmed by standard
ZEN	C18H22O5	ESI(−)	13.857	[M+CH3COO]−	377.1599	377.1606	-1.79	Authentic standard; RT; exact mass/adduct	Confirmed by standard
Zearalanone	C18H24O5	ESI(+)	13.727	[M+H]+	321.1689	321.1697	-2.34	Exact mass/adduct; RT; DB/literature consistency; no standard/MS/MS	Tentative
Zearalanone	C18H24O5	ESI(−)	12.858	[M−H]−	319.1559	319.1551	+2.51	Exact mass/adduct; RT; DB/literature consistency; no standard/MS/MS	Tentative
ZEN-14-glucuronide	C24H30O11	ESI(+)	17.473	[M+H]+	495.1856	495.1861	-0.99	Exact mass/adduct; RT; DB/literature consistency; no standard/MS/MS	Tentative
Zearalenol-O-glucoside	C24H34O10	ESI(+)	18.932	[M+H]+	483.2212	483.2225	-2.64	Exact mass/adduct; RT; DB/literature consistency; no standard/MS/MS	Tentative
Zearalenol-O-glucoside	C24H34O10	ESI(+)	18.932	[M+Na]+	505.2046	505.2044	+0.36	Exact mass/adduct; RT; DB/literature consistency; no standard/MS/MS	Tentative

Neutral exact masses used for calculations: ZEN, C18H22O5, 318.1467 Da; zearalanone, C18H24O5, 320.1624 Da; ZEN-14-glucuronide, C24H30O11, 494.1788 Da; zearalenol-O-glucoside, C24H34O10, 482.2152 Da.

Supplementary Figures

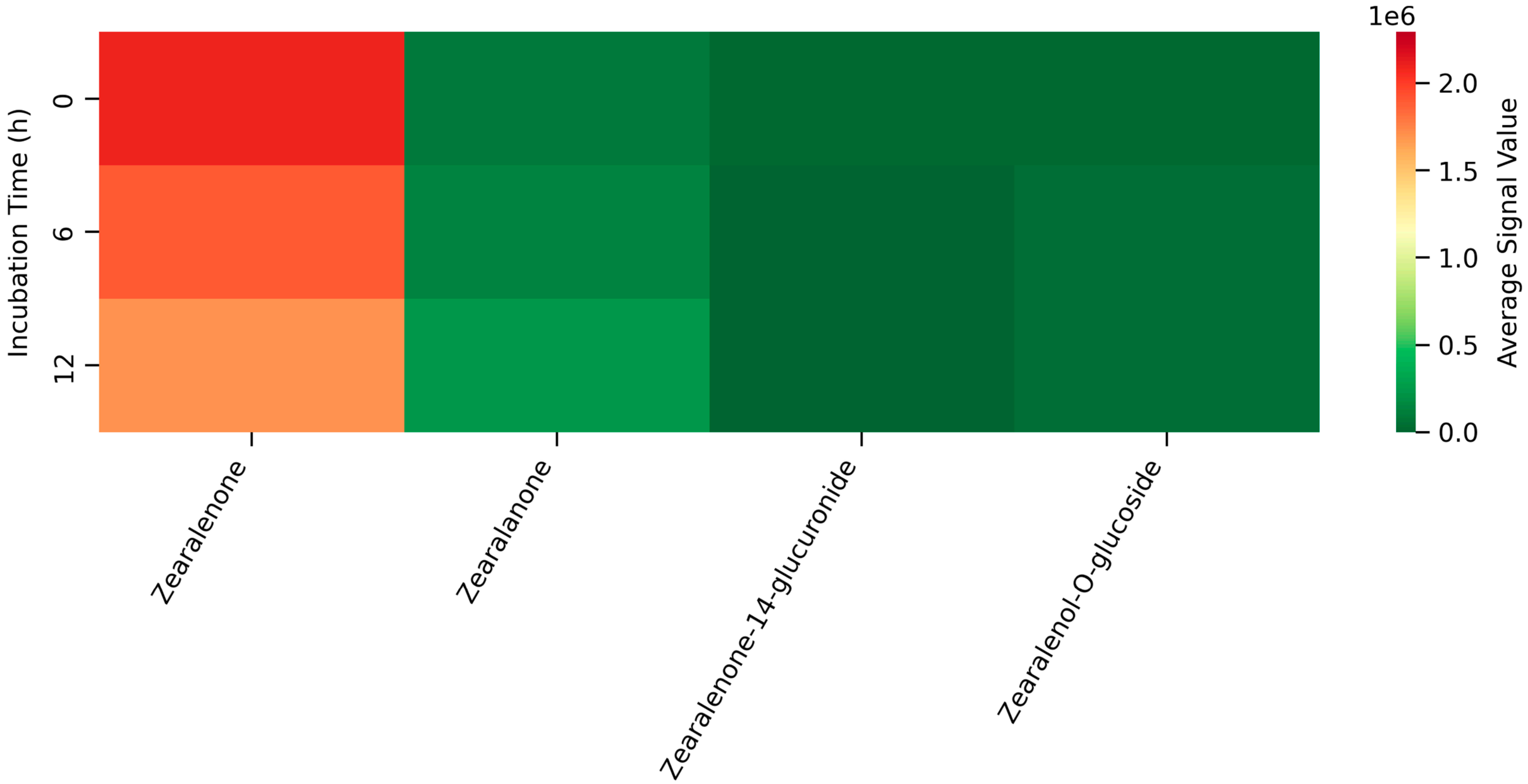

Figure S1. Heatmap of mean peak areas for ZEN and tentatively assigned transformation-related features during incubation at pH 3.50 in positive ionisation mode.

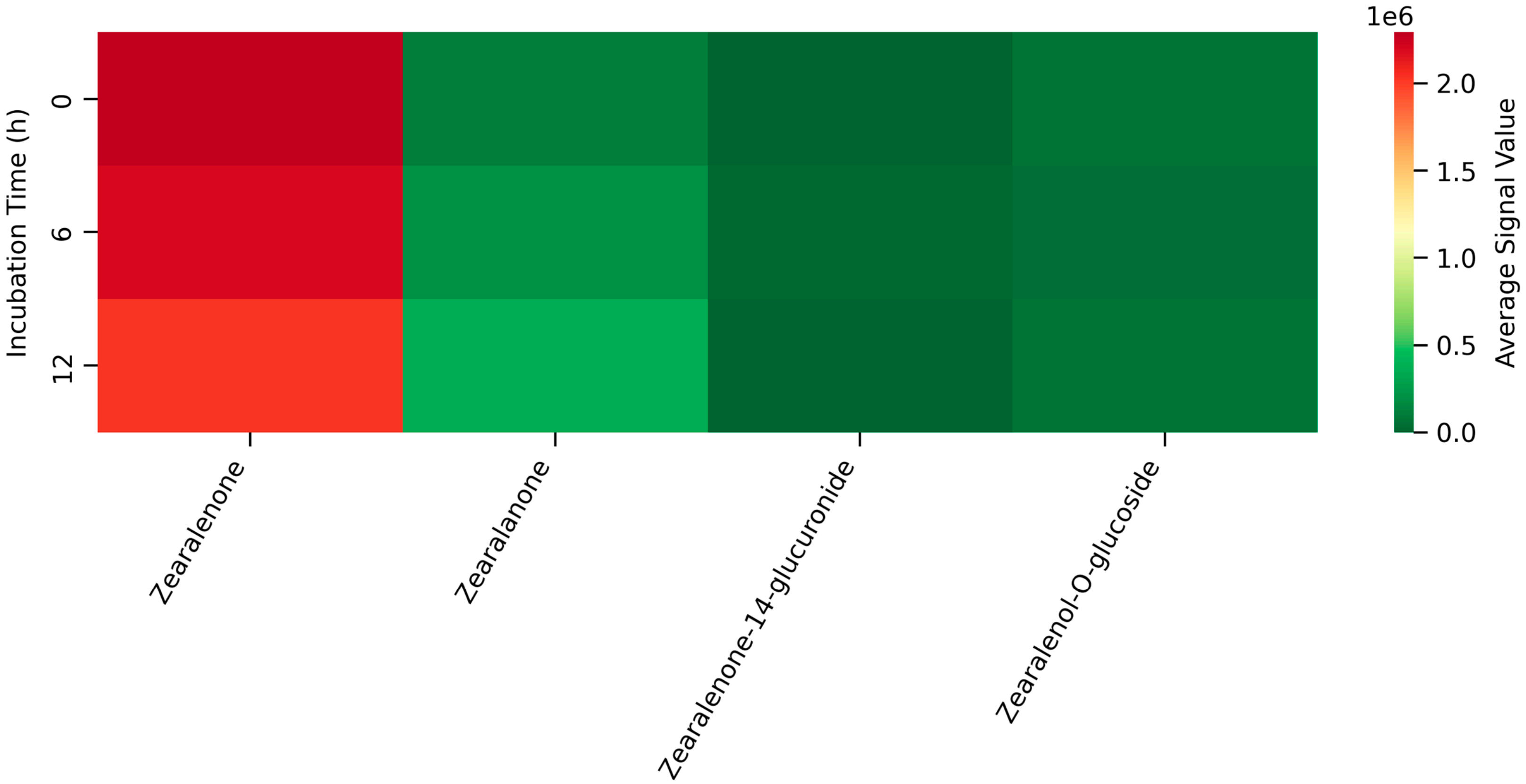

Figure S2. Heatmap of mean peak areas for ZEN and tentatively assigned transformation-related features during incubation at pH 7.00 in positive ionisation mode.

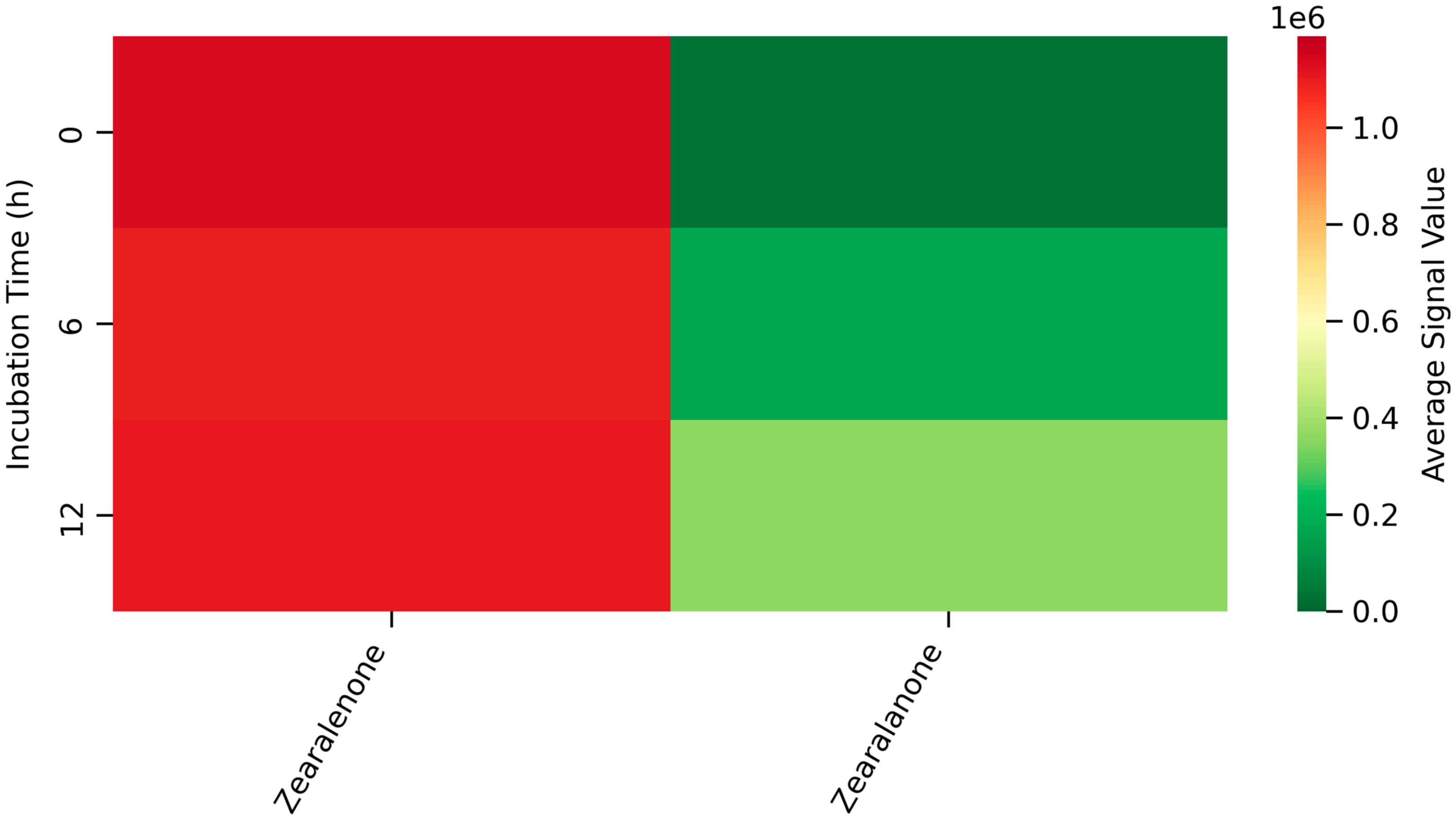

Figure S3. Heatmap of mean peak areas for ZEN and the tentatively assigned zearalanone-related feature during incubation at pH 3.50 in negative ionisation mode.

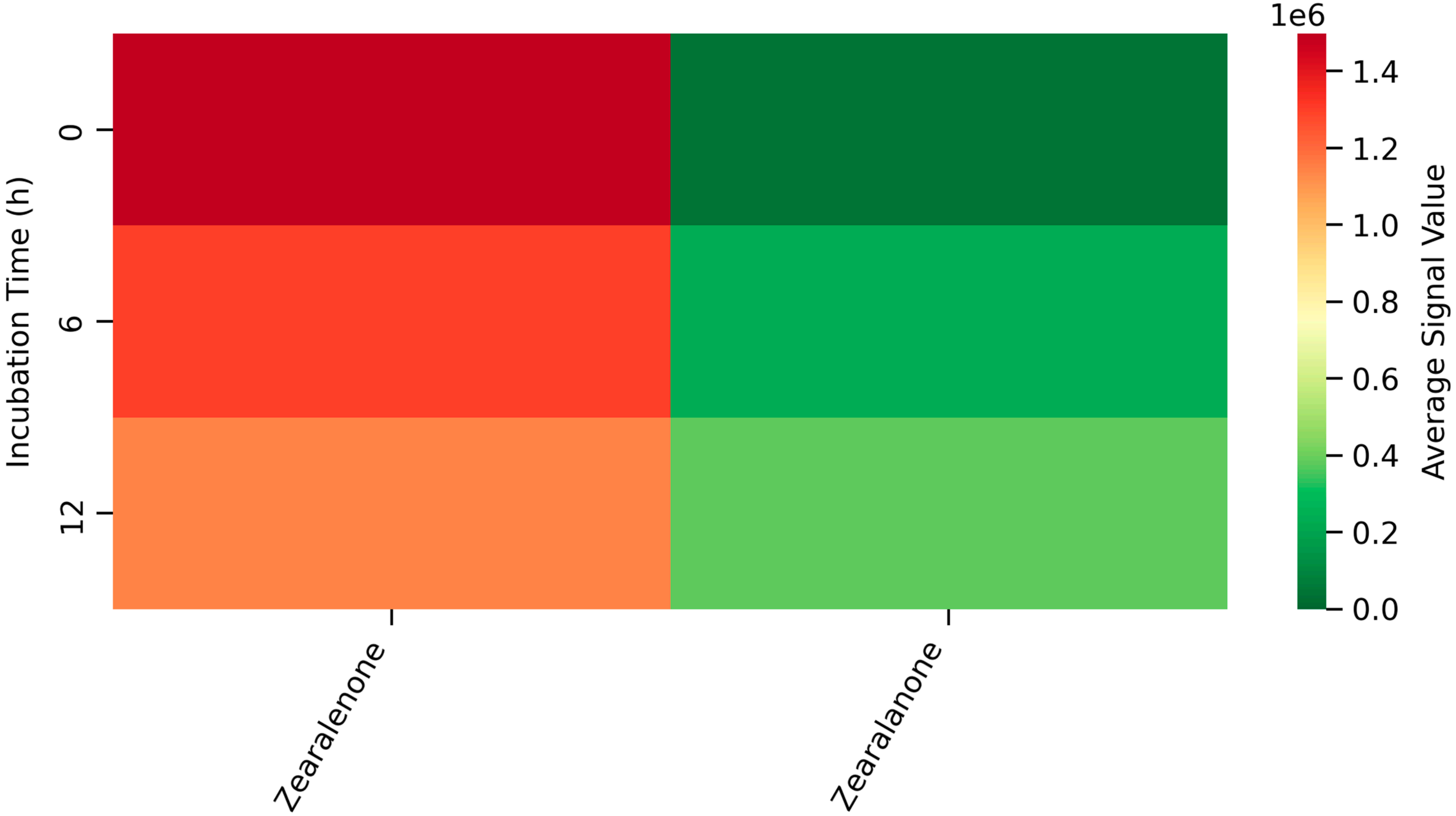

Figure S4. Heatmap of mean peak areas for ZEN and the tentatively assigned zearalanone-related feature during incubation at pH 7.00 in negative ionisation mode.
